# Fabrication of Ni-Rich 58NiTi and 60NiTi from Elementally Blended Ni and Ti Powders by a Laser Powder Bed Fusion Technique: Their Printing, Homogenization and Densification

**DOI:** 10.3390/ijms23169495

**Published:** 2022-08-22

**Authors:** Khashayar Khanlari, Qi Shi, Kefeng Li, Ke Hu, Chong Tan, Wen Zhang, Peng Cao, Inès Esma Achouri, Xin Liu

**Affiliations:** 1 Institute of New Materials, Guangdong Academy of Sciences, Guangzhou 510650, China; 2 Guangdong Provincial Key Laboratory of Metal Toughening Technology and Application, Guangzhou 510650, China; 3 National Engineering Research Center of Powder Metallurgy of Titanium & Rare Metals, Guangzhou 510650, China; 4 Département de Génie Chimique et de Génie Biotechnologique, Université de Sherbrooke, 2500 Boulevard de l’Université, Sherbrooke, QC J1K 2R1, Canada; 5 Department of Chemical and Materials Engineering, University of Auckland, Auckland 1142, New Zealand

**Keywords:** Ni-rich NiTi alloys, 58NiTi and 60NiTi, elemental Ni–Ti powder mixture, LPBF printing, HIP treatment, homogenization, densification

## Abstract

Compared to the equiatomic or near-equiatomic NiTinol alloys, Ni-rich NiTi alloys are suitable to be employed in structural applications as they exhibit higher hardness and are dimensionally stable. This research aimed to process two different grades of Ni-rich NiTi alloys, 58NiTi and 60NiTi, from Ni–Ti powder mixtures having about 58 wt.% and 60 wt.% Ni, respectively. This was performed by a laser powder bed fusion technique. At the first stage of this research, the printability of the used powder mixtures was investigated by applying different sets of printing parameters. Two appropriate sets were then selected to print the samples. Microstructural study of the printed parts revealed the existence of inhomogeneity in the microstructures. In addition, depending on the applied set of parameters, some amounts of cracks and pores were also present in the microstructure of these parts. Postprinting hot isostatic pressing procedures, performed at different temperatures, were developed to cause the reaction of phases, homogenize the parts, and possibly eliminate the existing flaws from the samples. Effects of these applied treatments on the microstructure, phase composition, density, dimensional integrity, and hardness of parts were sequentially studied. In essence, 58NiTi and 60NiTi parts having phase compositions complying with those of the equilibrium phase diagram were obtained in this research. However, the mentioned cracks and pores, formed in the microstructure of as-printed parts, could not be fully removed by postprocessing treatments.

## 1. Introduction

**Figure 1:**
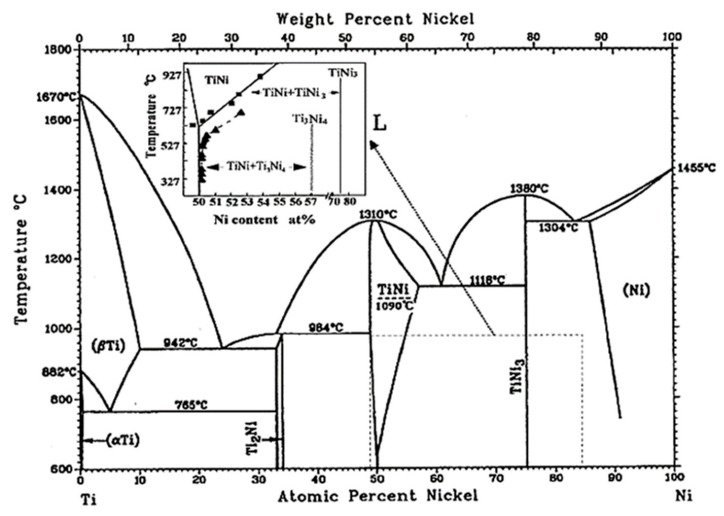
Binary NiTi phase diagram [1].

Ni-rich NiTi alloys show the possibility to dissolve an increased amount of Ni in NiTi composition by increasing the temperature above ~625 °C. In essence, the proportion of Ni_3_Ti, Ni_3_Ti_2_, and Ni_4_Ti_3_ Ni-rich phases, existing in the NiTi matrix of these alloys, decreases at temperatures above ~625 °C. Increasing the temperature and passing above the solutionizing temperatures, specific for each of these alloys, results in the formation of a single-phase microstructure in these parts containing solely NiTi (Figure 1) [1,2]. Under such conditions and once parts are cooled under very fast cooling rates, the formation of already dissolved Ni_3_Ti and Ni_3_Ti_2_ phases is prevented in the microstructure of these parts cooled to ambient temperatures. This is because, under such conditions, metastable nanoscale Ni_4_Ti_3_ particles, which their formation in the matrix is inevitable, do not have enough time to coarsen and decompose to Ni_3_Ti_2_ or stable Ni_3_Ti phases through the precipitation sequence of Ni_4_Ti_3_ → Ni_3_Ti_2_ → Ni_3_Ti [1,3,4,5,6].

The increased amount of Ni in NiTi composition plays a significant role in the phase transformation temperatures and shifts down the specific temperatures where austenitic NiTi is transformed to a martensitic one. In the specific case of 60NiTi, a very Ni-rich NiTi intermetallic containing ~60 wt.% (55 at.%) Ni and ~40 wt.% (45 at.%) Ti in its composition, the explained solutionizing and quenching procedure shifts down the phase transformation temperatures to well below room temperature (below −100 °C), making this alloy dimensionally stable for room temperature applications [1,7,8].

In addition, quenching 60NiTi from temperatures above ~1000 °C, where this intermetallic is of a single-phase microstructure containing solely NiTi, results in the generation of parts with a high hardness of ~60 HRC [9]. This amount of hardness is significantly higher than that of this alloy in as-processed conditions (~40 HRC). It is because of this reason that the discussed procedure is considered as a hardening treatment for this alloy system. Hornbuckle et al. [1] attributed the observed high hardness of solution-treated and quenched 60NiTi to the precipitation of a large volume fraction of nanoscale Ni_4_Ti_3_ platelets (~55 %) formed in the NiTi matrix. This level of Ni_4_Ti_3_ particle precipitation also leads to narrow austenitic NiTi matrix channels and a microstructural morphology ideal for an Orowan strengthening mechanism.

The well-studied equiatomic or near-equiatomic shape memory NiTinol alloys are not dimensionally stable, as their austenite–martensite phase transformation temperatures are around room temperatures and show a relatively low hardness value (~30 HRC), making them unsuitable for structural applications [8,10,11,12]. On the contrary, the combination of dimensional stability, a high hardness that is comparable to bearing steels, and other advantageous properties of 60NiTi make this Ni-rich grade of NiTi alloys a suitable candidate for structural applications [8,13,14,15,16]. As an example, this alloy is a good candidate to be employed as a rolling element bearing material that is highly corrosion resistant and endures high levels of shock loads before experiencing any permanent deformation, and also for lubricated sliding applications [17,18,19,20]. National Aeronautics and Space Administration (NASA) has developed an advanced hot isostatic pressing (HIP) manufacturing method to produce high quality/purity 60NiTi parts, devoid of oxide inclusions, stringers of oxides, and pores commonly observed in cast processed 60NiTi parts [5,21,22,23].

As DellaCorte et al. [10] explained, other very Ni-rich grades of NiTi alloys existing compositionally between equiatomic NiTi and 60NiTi, such as 58NiTi, 59NiTi, etc., might also show promising exploitable properties. The hardness values of these alloys in hardened conditions (~48 HRC for 58NiTi), though, are not significantly different than those of their furnace-cooled conditions (~40 HRC) and are remarkably lower than that of hardened 60NiTi (~60 HRC). This is because these alloys are less Ni-rich than 60NiTi and, consequently, contain a lesser amount of hardening nanoscale Ni_4_Ti_3_ in the NiTi matrix of their hardened parts. The amounts of generated Ni_4_Ti_3_ precipitates formed in the NiTi matrix of their hardened parts might not be sufficient to cause a notable change in the obtained hardness values as compared to those of their furnace-cooled conditions. In addition, contrary to what it is for hardened 60NiTi, the amounts of nanoscale Ni_4_Ti_3_ particles precipitated in the NiTi matrix of these alloys is less than the ideal amount needed for the activation of an Orowan strengthening mechanism [1].

Due to the well-discussed advantageous characteristics of printing processes, such as reducing the cost of machining and the capability to manufacture geometrically complex parts, a significant amount of research has been performed to print equiatomic and near-equiatomic NiTinol parts mainly from prealloyed powders and largely for biomedical and actuator applications. The effects of printing parameters on the phase formation, mechanical, phase transformation properties, and other characteristics of the developed parts are well-investigated and reported in the literature [24,25,26,27,28,29,30,31]. Printing of NiTinol parts from elementally mixed powders has been addressed to a much lesser extent as compared to the prealloyed ones. Once elementally mixed Ni–Ti powders are used, it is challenging to generate desirable intermetallic phases and avoid undesirable ones in parts produced from printing processes [24,32]. Shishkovsky and Gureev et al. [33,34,35], by employing a combination of self-propagating high-temperature synthesis (SHS) and selective laser sintering (SLS) in the same process, addressed the challenges to control different exothermic reactions leading to the generation of desirable intermetallic phases in porous parts processed from Ni–Ti powder mixtures.

However, the research work conducted on 3D printing of very Ni-rich grades of NiTi alloys, such as 58NiTi, 60NiTi, etc., with dense microstructures is not substantial. In a recent study, Khanlari et al. [36] printed parts from a Ni-rich Ni–Ti powder mixture, consisting of about 63 wt.% Ni and 37 wt.% Ti, by using a laser powder bed fusion technique (LPBF). The microstructure of the parts printed from a selected set of printing parameters (the set with which the used powder showed a good printability) was inhomogeneous and contained a significant amount of cracks. The inhomogeneity in the microstructure of parts was removed by applying an elementary HIP treatment, at 180 MPa and 1050 °C for 4 h, and a homogeneous microstructure containing desirable phases complying with the equilibrium binary NiTi phase diagram was obtained. It was understood that the cracks existing in the microstructure of the parts could be successfully replaced with some small pores by applying a secondary HIP treatment performed at the optimum temperature of 1129 °C, being just slightly above the melting start temperature of the printed samples.

The aim of the current research was to process dense and homogeneous 58NiTi and 60NiTi from elementally mixed powders by employing an LPBF method. The effects of different printing parameters on the printability of used powder mixtures were investigated at the first stage. In the next step, and after identifying the selected set of printing parameters with which the powder mixtures showed a good printability, the microstructures of the parts were investigated. The principles used in the recent study of Khanlari et al. [36] were then used as a guideline in this study to homogenize the microstructure of printed parts, eliminate the existing flaws, and improve their densities. The effect of the final hardening treatment on the microstructure, phase composition, and hardness of developed 58NiTi and 60NiTi parts were also studied. Differences observed in the processing of 58NiTi, 60NiTi, and 63NiTi (discussed in [36]), being different grades of Ni-rich NiTi alloys with different compositions, were also elaborated on in this study.

## 2. Results and Discussion

### 2.1. Powder Characteristics

**Figure 2:**
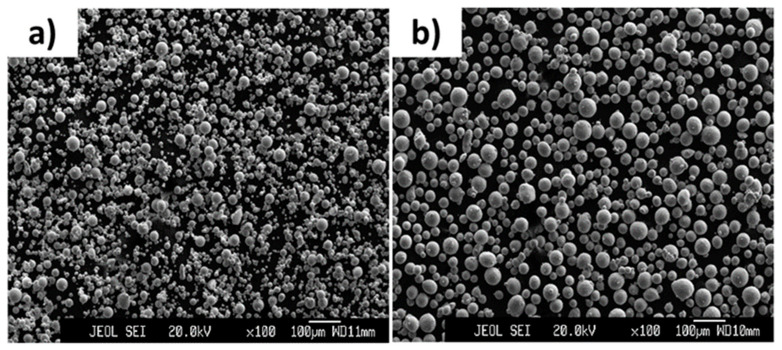
Images showing the microstructure of (**a**) Ni and (**b**) Ti powders.

Figure 2 shows the microstructures of purchased Ni (D90 = 43.3 µm) and Ti (D90 = 86.2 µm) elemental powders. As seen, the particles of both of the powders were mostly spherical with some satellites formed on them.

To obtain powder particles suitable for the printing process, the purchased powders were then sieved to below 53 µm. In the next step, sieved Ni and Ti powders were mixed for 16 h in a rotating mixer in two different Ni: Ti weight ratios of about 1.50:1.00 and 1.38:1.00. As a result of this, two different powder batches having compositions of, respectively, about 60 wt.% Ni + 40 wt.% Ti and 58 wt.% Ni + 42 wt.% Ti were obtained.

Some specific physical characteristics of powders, such as particle size distribution, Hall flowability, apparent density, and tap density are reported in Table 1. Since the compositions of powder batches were almost identical, the values for these physical characteristics were very similar together and for the sake of brevity, just the ones for the powder mixture having about 60 wt.% Ni + 40 wt.% Ti are reported here.

Table 2 reports the amounts of impurities existing in the powder mixtures. As seen, the powder mixtures, consisting of spherical particles, contained a low amount of impurity (~0.046 wt.%) and showed a particle size distribution having D90 < 50 µm (Table 1). As a result, the prepared powder mixtures were suitable to be used in the LPBF process.

### 2.2. Processing of the As-Printed Parts

A wide range of processing parameters, that included using laser powers ranging from 50 to 90 watts and scanning velocities of 200 to 600 mm/s, were employed in this research to print the samples. In all the different processing sets, the hatch spacing and layer thickness were kept constant at, respectively, 0.12 mm and 0.03 mm (Table 3).

The volumetric laser energy density inputs, hereinafter mentioned as energy input, were accordingly calculated based on the following classical equation:

E_V_ = P/(v × h × t) (1)

where E_V_ is the volumetric energy density input, P is the laser power, v is the scanning velocity, h is the hatch spacing, and t is the layer thickness.

Considering these, it can be concluded that the energy inputs ranging from 27.70 to 69.44 J/mm^3^ were employed in the processing of the printed samples. However, it should be noted that these calculated amounts of energy do not include the energy generated by different exothermic reactions that might have occurred during the synthesis process of parts from the powder mixtures. These exothermic reactions, caused by the chemical reactions that occurred between the elemental materials and different phases during the process, lead to additional energy inputs. The amount of these additional energy inputs depends on the synthesis conditions and the used processing parameters and might be different depending on the employed printing parameters [36].

Under conditions where the used laser power was 60 watts, the used powder mixtures showed a reduced printability as compared to the conditions where a laser power of 50 watts was employed to print the parts, even if the employed laser energy densities were very similar together (set 3 as compared to set 8 and set 4 as compared to set 9, Table 3). In addition, the powders did not show any printability with the employed laser powers of 70 and 90 watts. Similarly, the printability of the powders was reduced with the increase in volumetric laser energy densities. As an example, once the laser power of 50 watts was employed, the employed volumetric laser energy densities of 55.55 J/mm^3^ and 69.44 J/mm^3^ could not print the used powder mixtures (printing sets 6 and 7). In essence, the lower the used laser energy input is, the higher the number of printed cubic samples (out of 8).

The observed trends are similar to those observed in [36], in which a powder mixture with about 63 wt.% Ni + 37 wt.% Ti composition was used to print the parts. Considering these results, it can be interpreted that laser power plays the primary role in the successful printing of parts from the used Ni–Ti elemental powder mixtures and the laser energy input acts as the secondary role.

Residual stresses which are generated in the samples during the printing process might be the reason for the trends observed in the printability of parts. The level of generated residual stresses is affected by both the laser powers and laser energy inputs. It is estimated that the lower these factors are, the lower the temperature of the melt pool will be and, consequently, the lower the difference between the temperature of the melt pool and the plate on which the samples are printed. This sequentially leads to the generation of lower amounts of residual stresses in the printed samples as compared to the conditions where higher laser powers and laser energy inputs are used [24,27,37,38]. Note that the occurrence of phase transformations in the samples, the extent and type of which depend on different sets of processing parameters, can also be another source of residual stress generation in the samples. This factor is neglected in this study and its effects are not analyzed.

The high residual stresses that are formed in the first layers of the printing process result in a warping effect (i.e., separation of the sample from the build platform) in the processed parts. It is thought that this effect, once it is too high, can lead to the hit and removal of samples from the build platform by the movement of the blade depositing the next layer of powder. Consequently, the lower the used laser powers and energy inputs (Table 3), causing lower residual stresses and a less pronounced warping effect in the parts, the more the number of samples that could be printed from the employed powder mixtures.

**Figure 3:**
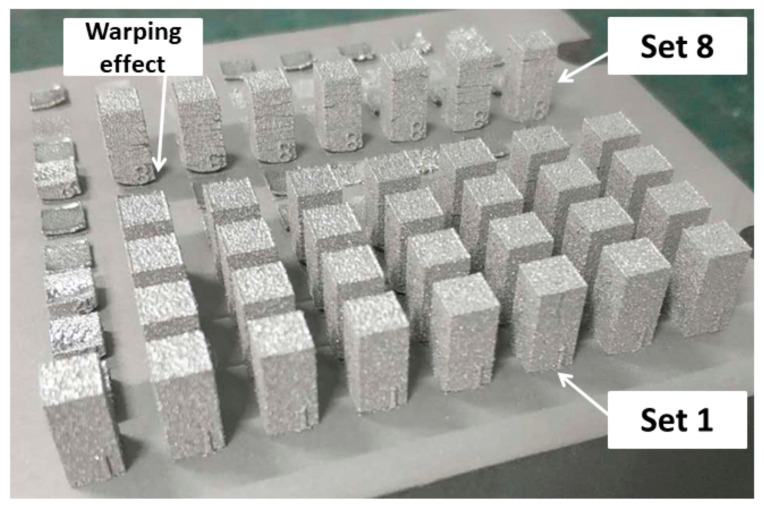
Image of samples, printed from about 58 wt.% Ni + 42 wt.% Ti powder mixture, processed by different processing sets.

It is interesting to note that the effect of generated residual stresses causing the warping effect is clear in the geometry of the printed samples. As observed in Figure 3, a more pronounced warping effect is observed in the samples (printed from about 58 wt.% Ni + 42 wt.% Ti powder mixture) processed by printing set 8, under which the samples were printed by a laser power of 60 watts, as compared to printing set 1 where the samples were printed under identical printing parameters but a laser power of 50 watts. The bottom of these samples is curvier than the ones processed by printing set 1.

In addition, it is noted that, once identical printing parameters were employed, 60 wt.% Ni + 40 wt.% Ti powder mixture showed a relatively better printability than the 58 wt.% Ni + 42 wt.% Ti one. In addition, 63 wt.% Ni + 37 wt.% Ti powder mixture showed a relatively better printability than the powder mixtures used in this study, once processed under identical processing conditions of those of sets 5 and 6 (Table 3) [36]. As seen in the binary NiTi phase diagram (Figure 1), the specific liquidus temperatures of the NiTi alloys containing ~55 wt.% Ni to ~65 wt.% Ni decrease as their composition becomes more Ni-rich. In essence, the temperature of the melt pool before passing the liquidus line for the parts printed from the powder mixture with 63 wt.% Ni was lower than that for those printed from the powder mixture with 60 wt.% Ni in its composition. Similarly, the temperature of the melt pool before passing the liquidus line for parts printed from the powder mixture having 60 wt.% Ni was lower than that of those printed from the powder mixture having 58 wt.% Ni in its composition. Consequently, amongst all the used powder mixtures, parts that were processed from 63 wt.% Ni + 37 wt.% Ti powder mixture experienced the lowest difference in the temperature of the melt pool before the start of the solidification process with that of the plate on which the samples were printed. The lower this difference and gap, the smaller the level of generated residual stresses and warping effects in the printed parts. These consequently explain the differences observed in the printability of these Ni-rich powder mixtures.

### 2.3. Characterization of the As-Printed Parts

**Figure 4:**
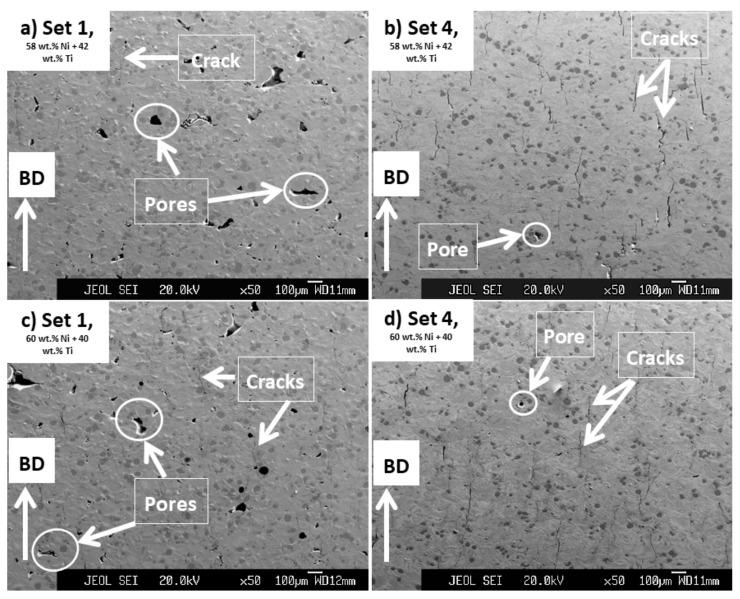
Images obtained from the cross-section of samples processed from powder mixtures having (**a**,**b**) 58 wt.% Ni + 42 wt.% Ti and (**c**,**d**) 60 wt.% Ni + 40 wt.% Ti compositions by processing sets 1 and 4 having energy inputs of 27.70 and 39.68 J/mm^3^, respectively. Some of the cracks and pores seen in the microstructure of these samples are shown in the images.

**Figure 5:**
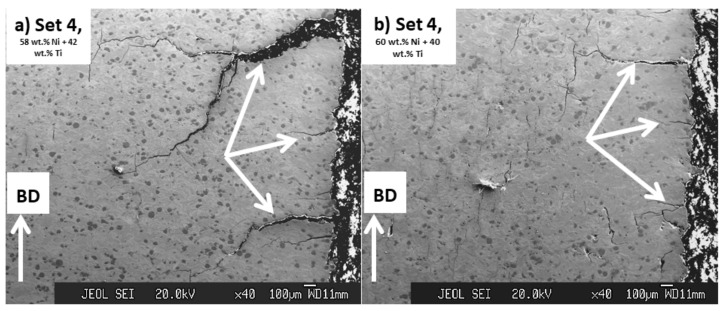
Images obtained from the cross-section of samples processed by processing set 4 having energy input of 39.68 J/mm^3^, from powder mixtures having (**a**) 58 wt.% Ni + 42 wt.% Ti and (**b**) 60 wt.% Ni + 40 wt.% Ti compositions. Some of the cracks seen in the corners of these samples are shown by arrows in the images.

The microstructures (Figure 4 and Figure 5) of the samples printed by sets 1 and 4 are discussed and analyzed here. The processing set 1 was chosen for further studies as the powder mixtures showed a perfect printability with this set and it had the lowest energy input (27.70 J/mm^3^) amongst all the other printing sets. Set 4 was selected as it had the highest energy input (39.68 J/mm^3^) that the powder mixtures showed an almost reasonable printability with (Table 3).

As seen in Figure 4, regardless of the composition of the powder mixtures, cracks and pores are readily observed in the microstructures of the printed parts. However, parts printed by set 1 were mostly consisting of pores (Figure 4a,c) and the ones printed by set 4 with many cracks formed in parallel to the BD (Figure 4b,d). In addition, it is noted that the pores observed in the microstructures of parts printed by set 1 were larger than those existing in parts processed by set 4 and some of these pores had irregular geometries being almost perpendicular to BD.

It is expected that the cracks were formed as a result of the generation of significant amounts of residual stresses in the printed samples. As the parts printed by set 4 were processed using a higher energy input than the ones with set 1, a larger level of residual stresses and, consequently, cracks were introduced in their microstructures as compared to the set 1 printed ones [27]. Small pores seen in the microstructures of parts might have roots in the gases which got mixed with the powder mixtures during their consolidation process. These gases then got trapped within the molten pool that was formed during the printing process causing the formation of the observed pores [39]. On the other hand, irregular larger pores, seen mainly in the microstructure of the parts processed by set 1, were probably the effects of a lack of bonding and diffusion between the printed layers having roots in the low energy input (27.70 J/mm^3^) used to process these parts [24].

As seen in Figure 5, in addition to cracks formed in parallel to BD, large cracks were also seen in the microstructure of parts printed by set 4. These cracks were initiated from the corner of the printed parts and were almost perpendicular to the BD. These cracks were absent in the microstructure of parts that were processed by set 1. In addition, it is noted that these types of cracks existing in the microstructure of parts printed from 58 wt.% Ni + 42 wt.% Ti powder mixture were more aggressive (Figure 5a) than those in parts processed from 60 wt.% Ni + 40 wt.% Ti (Figure 5b).

These observations are another evidence for the existence of a higher level of residual stress in parts processed by set 4 (39.68 J/mm^3^) than those printed by set 1 (27.70 J/mm^3^) and also in the ones processed from 58 wt.% Ni + 42 wt.% Ti powder mixture than those printed from the 60 wt.% Ni + 40 wt.% Ti powder. These results once again emphasize the role of printing energy input and the temperature of the melting pool before the solidification in the generation of residual stresses in the printed parts studied in this research.

**Figure 6:**
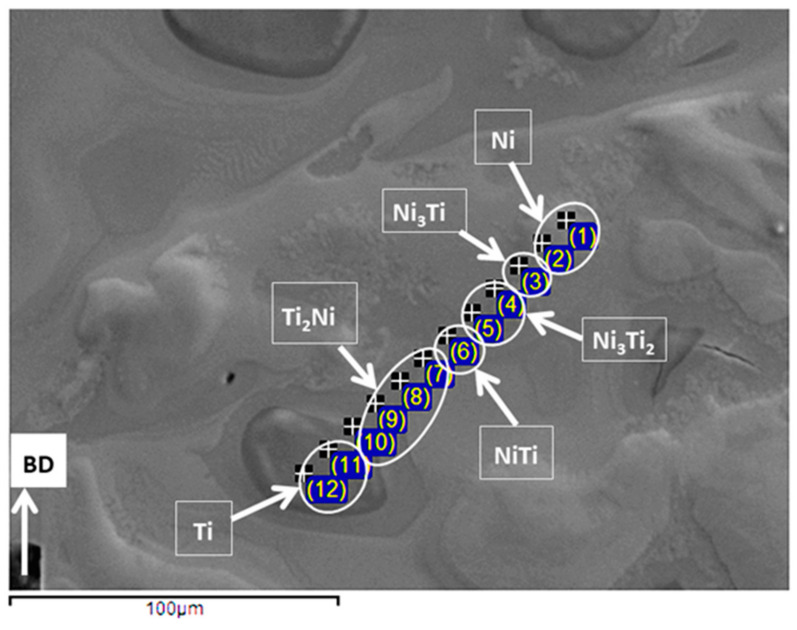
Results of EDS elemental ratio studies conducted on samples printed by set 4 and processed from 60 wt.% Ni + 40 wt.% Ti powder mixture.

Regarding the phase composition of printed samples, the microstructures of all the samples were similar together and the applied printing processes did not lead to a complete reaction between the elemental Ni and Ti existing in the powder mixtures. Figure 6 shows the results obtained from EDS studies and the existence of an inhomogeneous microstructure containing Ni-rich and Ti-rich areas is clear in the processed samples. Note that the identified phases were interpreted based on the Ni: Ti ratio reported for different NiTi phases in [22]. Ti-rich regions are darkest under the SEM images (Figure 4, Figure 5 and Figure 6) and the results show that they contained about 1–5 wt.% Ni in their composition, suggesting the formation of βTi. The areas between the Ni-rich and Ti-rich regions, which appear to be the original cores of the Ti and Ni elemental particles used in the powder mixtures, were surrounded by Ti_2_Ni, NiTi, Ni_3_Ti_2_, and Ni_3_Ti in a gradient mode. These results are in accordance with the findings of previous research and the one conducted on 63 wt.% Ni + 37 wt.% Ti powder mixture [26,36]. The formation of undesired intermetallic phases, pure nickel, and pure titanium is typical in parts processed from elementally blended powders.

### 2.4. Microstructural Homogenization and Densification

Considering the observed microstructures of the as-printed parts, an elementary HIP treatment was designed and conducted on these parts to homogenize the phase composition of the as-printed samples and eliminate the flaws, namely pores and cracks, observed in the microstructures of these parts. In this HIP treatment, samples were kept at 1050 °C for a long duration of 4 h and under a pressure of 180 MPa. Other details regarding the applied processing parameters, such as heating rate, etc., for this process are explained in Section 2.2—LPBF processing and HIP treatment. This treatment was identical to that applied in our previous study on as-printed parts, processed from 63 wt.% Ni + 37 wt.% Ti powder mixture, having an inhomogeneous microstructure. Considering the obtained results reported in [36], this treatment resulted in the homogenization of as-printed parts but was not successful in eliminating the existing cracks and pores.

In essence, the reason for choosing this HIP temperature was that it is above 942 °C where a eutectic liquid (Figure 1) is estimated to be generated amongst β-Ti phase and Ti_2_Ni intermetallic, formed around β-Ti in the as-printed samples (Figure 6) [40,41,42,43]. It is thought that the generation of this liquid will enhance the homogenization process as, under such conditions, the reaction rate amongst different phases is expected to be increased [36,44].

**Figure 7:**
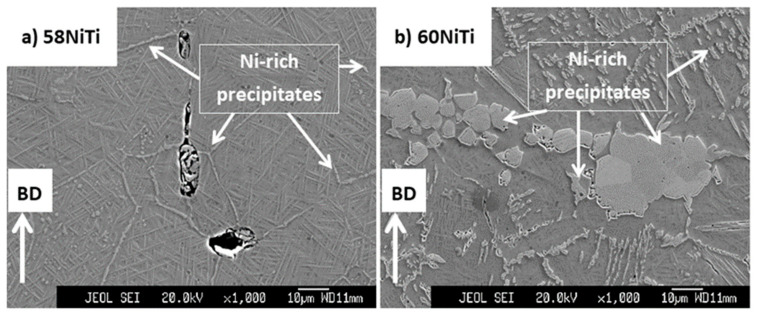
Images obtained from the cross-section of HIP-treated (at 1050°C for 4 h under an applied pressure of 180 MPa) samples being printed from (**a**) 58 wt.% Ni + 42 wt.% Ti and (**b**) 60 wt.% Ni + 40 wt.% Ti powders mixtures. Microstructures complying with those of (**a**) 58NiTi and (**b**) 60NiTi, respectively, were obtained as a result of this treatment. Some of the Ni_3_Ti and Ni_3_Ti_2_ Ni-rich phases seen in the microstructure of these samples are shown in the images.

As seen in Figure 7, the microstructures of the HIP treated parts, regardless of the applied processing sets used to print the treated parts, appear homogeneous, and as the EDS analysis shows, Ni-rich phases, namely, Ni_3_Ti and Ni_3_Ti_2_, were precipitated in NiTi matrix. These microstructures comply with what is expected from the NiTi binary phase diagram and are similar to the microstructures of reference Ni-rich N-Ti parts shown in the literature [16]. Based on the NiTi binary phase diagram, Ni_3_Ti and NiTi are stable phases for these specific compositions. However, metastable Ni_3_Ti_2_ and Ni_4_Ti_3_ can also precipitate in the NiTi matrix of treated parts upon slow cooling from the high temperatures that the HIP treatment was applied at [1,36].

**Figure 8:**
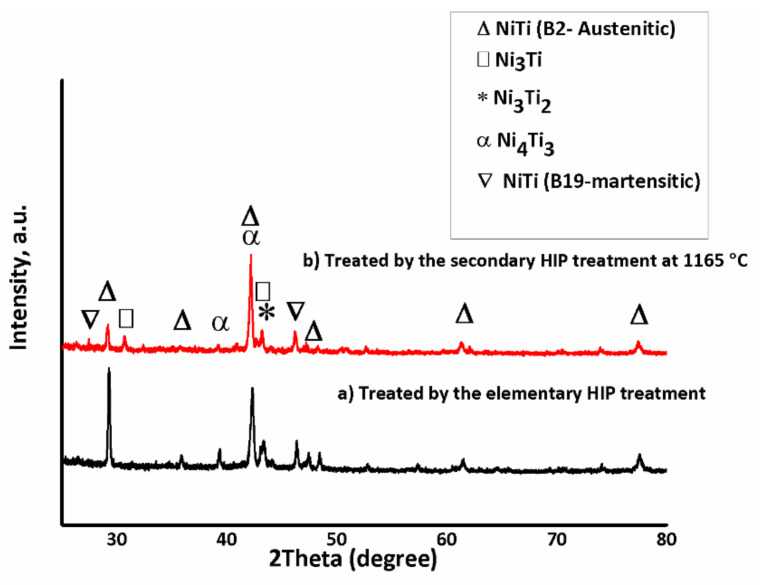
XRD patterns of (a) 60NiTi samples processed by elementary HIP treatments, from parts printed by set 4 having an energy input of 39.68 J/mm^3^ and (b) the same 60NiTi samples treated by the secondary HIP treatment conducted at 1165 °C for 1 h and under an applied pressure of 180 MPa.

These findings were further confirmed by the XRD tests (Figure 8a). Note that for the sake of brevity, just the HIP-treated 60NiTi samples processed by set 4, having an energy input of 39.68 J/mm^3^, are shown here. In essence and as confirmed by the microstructural and EDS studies, the microstructures of these furnace-cooled parts were consisting of Ni-rich phases precipitated in the NiTi matrix. Moreover, no evidence of elemental Ni and Ti, existing in the microstructure of the as-printed parts, was present in the patterns. NiTi was consequently formed in both austenitic and martensitic structures, as the formation of Ni-rich phases results in the depletion of Ni from the surrounding NiTi matrix areas leading to the compositional differences in this phase [45]. As a result of these investigations, it was concluded that the discussed HIP treatment resulted in the generation of 60NiTi and 58NiTi, respectively, from parts printed from 60 wt.% Ni + 40 wt.% Ti and 58 wt.% Ni + 42 wt.% Ti powder mixtures.

**Figure 9:**
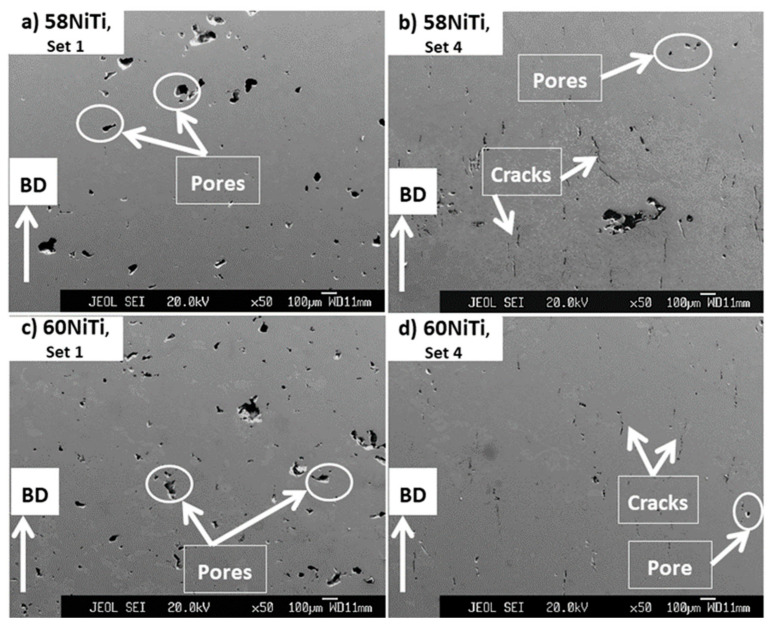
Images obtained from the cross-section of (**a**,**b**) 58NiTi and (**c**,**d**) 60NiTi samples processed by elementary HIP treatments from parts printed by sets 1 and 4 having energy inputs of 27.70 and 39.68 J/mm^3^, respectively. Some of the cracks and pores seen in the microstructure of these samples are shown in the images.

In terms of existing flaws such as cracks and pores, the obtained microstructures, despite being compositionally very different as compared to their as-printed conditions (Figure 4, Figure 5 and Figure 6), were very similar to the as-printed ones. As presented in Figure 9, the characteristics of flaws existing in the microstructure of each HIP-treated part were similar to its as-printed condition. In essence, cracks and pores were present in the microstructures of both 58NiTi and 60NiTi parts. The microstructures of parts obtained from those printed by set 1 were dominated by large pores; the ones processed from those printed by set 4, with cracks.

**Figure 10:**
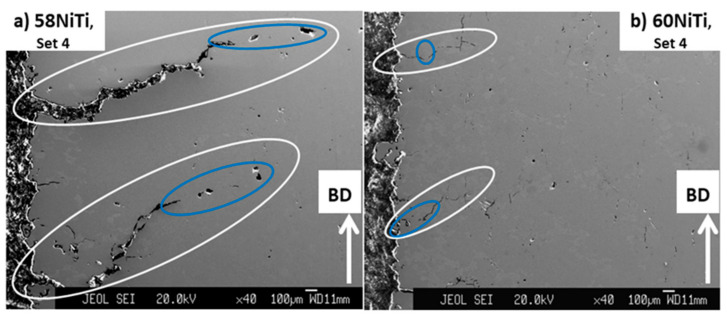
Images obtained from the cross-section of (**a**) 58NiTi and (**b**) 60NiTi parts processed from parts printed by processing set 4 having an energy input of 39.68 J/mm^3^. The white ovals show the total length of cracks, and the blue ones show the areas where the cracks were thought to be closed as a result of the applied HIP treatment.

However, a more careful observation in the microstructures shows that the HIP treatments were apparently successful in closing a portion of the large radial cracks propagated from the corner of parts printed by set 4 (Figure 5). As seen in Figure 10, the areas marked with the white ovals appear to be the initial cracks that existed in the as-printed parts and the blue ones show the portion which is thought to have been closed due to the effect of applied HIP treatment.

Under conditions where the temperature is increased above about 625 °C (Figure 1), Ni-rich NiTi alloys can dissolve an increasing amount of Ni in the NiTi phase [1]. Melting of the NiTi phase is expected to occur as the temperature is increased further and it approaches the solidus line. Parts will consequently become fully melted once the temperature passes the liquidus line. Considering the NiTi binary phase diagram (Figure 1) and for Ni-Ti alloys containing ~55 wt.% Ni to ~65 wt.% Ni, each specific Ni-rich NiTi alloy has specific solidus and liquidus temperatures and these temperatures are lower for Ni-rich NiTi alloys than less Ni-rich ones. The existing gap between the solidus and liquidus temperatures of these Ni-rich NiTi alloys provides an opportunity for their densification. In essence, it is expected that treating the parts in the temperature range between their solidus and liquidus lines leads to the generation of liquid in the samples. This sequentially increases their malleability, as compared to the conditions that the samples are in their solid-state phase. This condition combined with the applied pressure of the HIP process is expected to lead to the densification of parts. However, caution needs to be applied to the temperatures where the samples are treated, as too high temperatures, even if below liquidus line, cause the generation of an excessive amount of liquid in the parts, deteriorating their dimensional integrity.

Following this strategy and after applying the secondary HIP treatment at 1129 °C, the cracks existing in the microstructure of the 63NiTi parts, studied in our previous study [36], were replaced with some small pores. This temperature was the most effective and the highest one amongst the tested temperatures that did not deteriorate the dimensional integrities of the parts. The dimensional integrities of the parts were affected at 1131 °C, being slightly above 1129 °C [36].

Considering this, secondary HIP treatments were designed and applied at different temperatures on the homogenized 58NiTi and 60NiTi samples processed in this study. Note that for each of the applied treatments, the HIP procedures were performed for 1 h and under an applied pressure of 180 MPa. The details regarding the other processing parameters were identical to those of elementary HIP treatments and are explained in Section 2.2—LPBF processing and HIP treatment. These HIP treatments were initially performed at 1180 °C, a temperature chosen randomly and being certainly above the solidus temperatures of 58NiTi and 60NiTi alloys; based on the equilibrium NiTi binary phase diagram (Figure 1). Based on the obtained results, the HIP temperatures applied on each set of samples were varied around 1180 °C. These were conducted to identify the maximum HIP temperature at which the dimensional integrities of treated 60NiTi and 58NiTi parts were respected. Note that the observed conditions and effects of different temperatures on the dimensional integrities of parts are valid for the samples studied in this research being treated by the HIP process and do not follow the equilibrium condition determined in Figure 1.

Considering the results presented in Table 4, 1165 °C, 1175 °C, and 1170 °C were the maximum temperatures at which the dimensional integrities of 60NiTi and 58NiTi parts that were processed from the samples obtained by printing sets 1 and 4 were respected, respectively. In addition, it is noted that the maximum temperatures at which the dimensional integrities of 58NiTi parts were respected were relatively higher than those of 60NiTi and those for 60NiTi was higher than that of 63NiTi studied in our previous study (~1129 °C) [36]. These are because, as explained, the specific liquidus and solidus temperatures of the NiTi alloys containing ~55 wt.% Ni to ~65 wt.% Ni decrease as their composition becomes more Ni-rich. As a result, a higher temperature is required to deteriorate the dimensional integrity of 58NiTi than what is needed for 60NiTi and similarly for 60NiTi as compared to 63NiTi.

**Figure 11:**
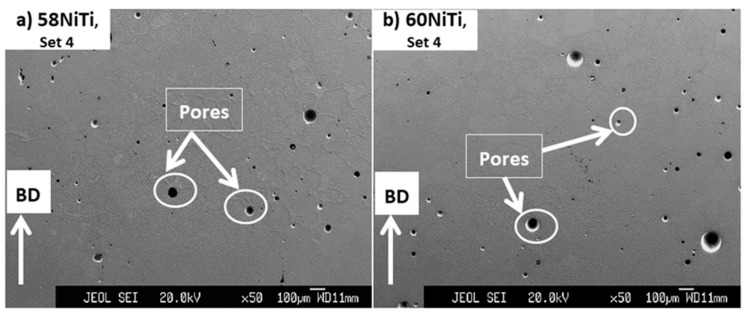
Images obtained from the cross-section of secondary HIP-treated (**a**) 58NiTi and (**b**) 60NiTi samples processed from parts printed by set 4 having an energy input of 39.68 J/mm^3^. The secondary HIP treatments for 60NiTi parts were conducted at 1167 °C and for 58NiTi ones at 1175 °C. Some of the pores seen in the microstructure of these samples are shown in the images.

Under conditions that the secondary HIP treatments were performed at temperatures at which the dimensional integrities of parts were affected, a noticeable change was observed in the microstructure of the parts. As seen in Figure 11, applying the secondary HIP treatments on 60NiTi and 58NiTi parts, printed by set 4 and homogenized by elementary HIP processes, at 1167 °C and 1175 °C, respectively, could successfully eliminate the cracks existing in the microstructure of these parts (Figure 9) and replace them with some pores (Figure 11). These are probably because, once treated at the mentioned temperatures, too much liquid was generated in these parts. As presented in Table 4, the parts lost their dimensional integrities under treatments conducted at these temperatures, taking them out of consideration for practical applications.

**Figure 12:**
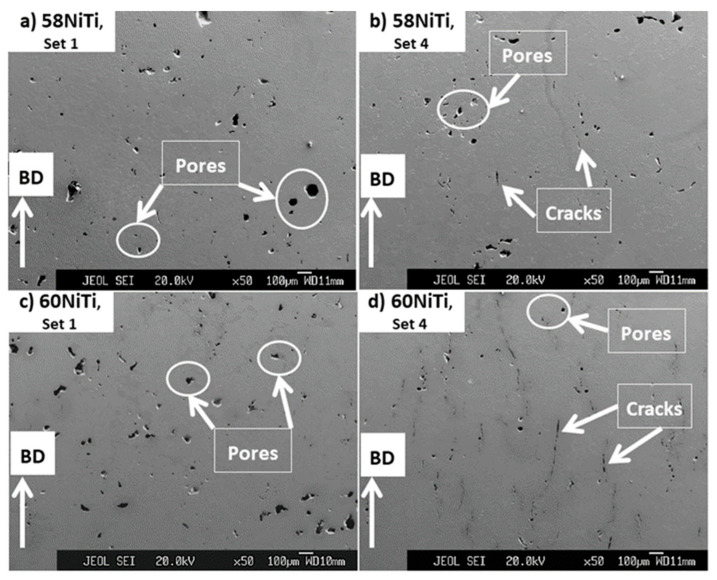
Images obtained from the cross-section of secondary HIP-treated (**a**,**b**) 58NiTi and (**c**,**d**) 60NiTi samples processed from parts printed by sets 1 and 4, having energy inputs of 27.70 and 39.68 J/mm^3^, respectively. The secondary HIP treatments for 60NiTi parts were conducted at 1165 °C and for 58NiTi ones printed by sets 1 and 4 at 1175 °C and 1170 °C, respectively. Some of the cracks and pores seen in the microstructure of these samples are shown in the images.

Figure 12 shows the microstructural images of parts treated at the temperatures at which the dimensional integrities were respected (highlighted in Table 4). As seen, the application of secondary HIP treatments at the discussed temperatures did not cause any noticeable change in the microstructural defects of 60NiTi and 58NiTi parts, obtained after applying the elementary HIP treatments on the as-printed parts processed by printing sets 1 and 4 (Figure 9). This might be because not much liquid was generated in the treated parts at these temperatures and samples were not malleable enough for the elimination of the existing flaws. These corroborate the results of density measurements reported in Table 5. As seen, the relative densities of the secondary HIP-treated parts (ranging between ~94 and ~97%) were almost identical to their elementary HIP-treated conditions (ranging between ~93 and ~98%).

In addition, the obtained relative densities appeared to be independent of the compositions of the printed parts. Instead, it is noted that the relative densities of these furnace cooled parts were mainly dependent on the energy input of the processing sets used to print them. As expected, parts processed from those printed by processing set 4, having an energy input of 39.68 J/mm^3^, were relatively denser than parts obtained from those printed by processing set 1, having a lower energy input of 27.70 J/mm^3^ (Table 5). This is because the microstructures of parts printed by set 1 were mainly dominated by large pores; whereas, those of parts printed by set 4 were mainly containing cracks.

To understand the effects of printing and postprinting treatments on the impurity contents of parts as compared to those of powder mixtures, the amounts of N and O existing in the compositions of samples processed under the following conditions were measured: (1) as-printed parts processed by set 4 and using the 58 wt.% Ni and 42 wt.% Ti powder mixture; (2) 58NiTi parts, obtained after applying the elementary HIP treatment on these as-printed parts; and (3) 58NiTi parts, obtained after applying secondary HIP treatments at 1170 °C on these elementary HIP-treated ones (Table 6).

It was noted that after printing the powder mixture, the amount of O increased from ~0.0380 wt.% to ~0.0405 wt.% (Table 1 and Table 6). In a similar trend, applying elementary and secondary HIP treatments increased the amounts of O and N existing in the compositions and, amongst all the processed parts, the secondary HIP-treated ones contained the highest amounts of impurities (Table 6). These make sense as the liquid was generated in parts in each of these different processing steps. In the printing step, the powder mixtures were melted and then got solidified. As mentioned, a eutectic liquid was expected to be generated in the microstructures during the conduction of elementary HIP treatments on the as-printed parts at 1050 °C. Similarly, applying the secondary HIP treatments resulted in the partial melting of the NiTi phase and the generation of liquid in the treated parts. It is normal that the O and N, existing in the atmosphere of the printing machine and HIP furnace, reacted with the generated liquids causing the observed increase in the amounts of impurities. Since oxygen and nitrogen are almost insoluble in the NiTi phases, it is thought that Ti_4_Ni_2_X_y_ (X=O, N) particles were also formed in the microstructure of the homogenized 58NiTi and 60NiTi parts [45]. However, the absence of a sharp peak, attributed to these phases, in the XRD patterns of HIP-treated parts (Figure 8) can be interpreted as evidence implying that the amounts of these generated particles were not significant. Considering the XRD results (Figure 8), the phase compositions of 58NiTi and 60NiTi parts treated by secondary HIP treatments were identical to their elementary HIP-treated conditions.

**Figure 13:**
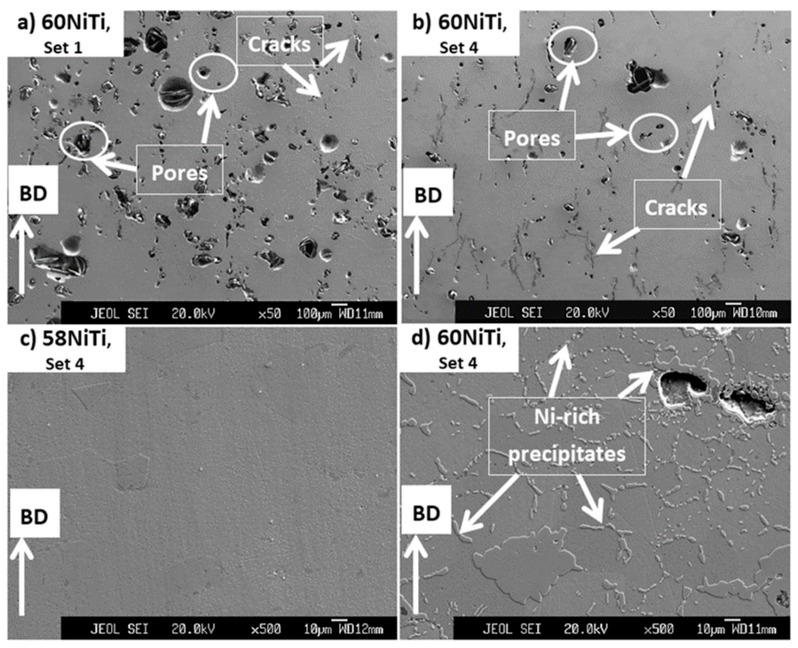
Images obtained from the cross-section of hardened secondary HIP-treated (**a**,**b**,**d**) 60NiTi and (**c**) 58NiTi samples processed from parts printed by (**a**) set 1 and (**b**–**d**) set 4 having energy inputs of 27.70 and 39.68 J/mm^3^, respectively. The secondary HIP treatments for 60NiTi parts were conducted at 1165 °C and for 58NiTi at 1170 °C.

To understand the effects of hardening treatment on the microstructures and properties of parts processed in this research, these secondary HIP-processed 58NiTi and 60NiTi parts were heated to 1000 °C in a muffle furnace, held at this temperature for 1 h and then were water quenched. Images related to the hardened 60NTi parts are shown here and as seen (Figure 13a,b), hardening treatments resulted in an increase in the amounts and the sizes of pores existing in the microstructure of secondary HIP-treated parts (Figure 12c,d). In addition, due to the effects caused by these heat treatments, it appears that the already existing cracks got relatively wider in size. As noted, the average relative densities of these parts decreased consequently (Table 5). Expansion and increase in volume in parts caused by heating the parts to a high temperature of 1000 °C and their subsequent quenching might be a reason for the occurrence of this phenomenon. Another reason might be attributed to the physical swelling of the parts having roots in the expansion of gas, trapped in the residual pores, during subsequent muffle furnace heating. These, consequently, caused the expansion of the micro- and macrosize pores existing in the microstructure of secondary HIP-treated parts [46]. A similar trend was observed for the 58NiTi parts.

As discussed before, due to the effects of using a lower energy input in the processing of parts printed by set 1 as compared to set 4 processed ones, parts processed by set 1 already had more amounts of porosity with larger sizes than set 4 processed samples (Figure 12). These led to the more pronounced, discussed effect of hardening treatment, on the characteristics of the existing flaws, on parts processed from set 1 than set 4 processed ones (Figure 13a,b). Consequently, while the decrease in the relative densities of set 4 printed parts was not major (about 3–4%), conduction of hardening treatment led to an about over 10% decrease in relative densities of the set 1 processed ones (Table 5).

Moreover, it was noted that, while the microstructures of the hardened secondary HIP-treated 58NiTi parts were devoid of Ni_3_Ti_2_ and Ni_3_Ti Ni-rich precipitates, the microstructures of the hardened 60NiTi parts still consisted of Ni_3_Ti and Ni_3_Ti_2_ phases (Figure 13c,d), even though it is to a lesser extent than those of furnace-cooled ones (Figure 7b). Considering these, it can be concluded that the applied hardening treatments, while being successful in the dissolution of Ni_3_Ti_2_ and Ni_3_Ti Ni-rich precipitates existing in the microstructure of secondary HIP-treated 58NiTi parts, were not successful in fully dissolving or preventing the partial precipitation of these phases in the matrix of 60NiTi ones.

It is expected that the porosities and cracks existing in the microstructures of these parts acted as an insulator during the quenching process causing the consequent decrease in the cooling rate [46]. As the flaw characteristics of 58NiTi and 60NiTi parts processed in this research were almost identical together, it is expected that, for both types of these samples, the effects of these flaws on the generated cooling rate were in the same range. Considering that it is expected that both 60NiTi and 58NiTi parts were fully solutionized at 1000 °C, the generated cooling rate, apparently being adequate to stop the precipitation of Ni_3_Ti_2_ and Ni_3_Ti in the matrix of 58NiTi parts, provided enough time for some of the Ni_4_Ti_3_ particles to go through the Ni_4_Ti_3_ → Ni_3_Ti_2_ → Ni_3_Ti precipitation sequence during the cooling process of solutionized 60NiTi parts. These results suggest that a higher cooling rate is needed for stopping the precipitation of Ni_3_Ti_2_ and Ni_3_Ti Ni-rich precipitates in the NiTi phase for the 60NiTi alloy system as compared to that of the 58NiTi alloy system. This might be because 60NiTi is more Ni-rich than 58NiTi, and as a result, there is a higher tendency for the fast precipitation of Ni_3_Ti_2_ and Ni_3_Ti Ni-rich precipitates in the NiTi matrix of this alloy system than that of 58NiTi.

Table 5 includes the average hardness values of the elementary HIP-treated, secondary HIP-treated and hardened 58NiTi and 60NiTi parts. As seen, the average apparent hardness values of the secondary HIP-treated parts (ranging from ~38.8 to ~46.1 HRC) were in the same range as their elementary HIP-treated conditions (ranging from ~42.3 to ~44.8 HRC). In addition, no meaningful correlations were noted amongst the hardness values, the composition of parts, and the processing sets used to print them. This might be because the relative densities of these entire elementary and secondary HIP-treated parts were almost in the same range. In addition, we know that the hardness values of furnace-cooled dense NiTi alloys, having different compositions, are not significantly different as compared to each other [47].

Conduction of hardening treatments on the secondary HIP-processed parts led to a significant decrease in the hardness values of parts printed by set 1 from a range between ~38.9 and ~42.4 HRC to a range between ~19.6 and ~26.1 HRC. This makes sense as the relative densities of these parts were also significantly decreased (about over 10%) after the conduction of hardening treatments (Table 5). Despite the successful solution of Ni_3_Ti and Ni_3_Ti_2_ phases in the NiTi matrix of 58NiTi parts, the hardness of these parts processed by set 4 was also decreased, from ~46.1 HRC measured in the secondary HIP ones to ~35.8 HRC obtained after applying the hardening treatment. This can be attributed to the decrease observed in the relative density of these parts from ~97% to ~93% and the widening of cracks that occurred in their microstructures caused by the hardening treatment. However, the conduction of hardening treatments on 60NiTi parts printed by set 4, despite not being fully successful in the dissolution of Ni_3_Ti and Ni_3_Ti_2_ phases in the NiTi matrix and causing a drop in the relative densities of these parts from ~97% to ~94%, led to the increase in hardness from ~38.8 HRC, measured in the secondary HIP treated parts, to ~45.9 HRC. These results emphasize the higher hardening potential of the 60NiTi alloy system as compared to that of the 58NiTi one. As reported in the literature, the hardness of dense 58NiTi alloy, despite being similar to that of 60NiTi in furnace-cooled conditions, is significantly lower (~48 HRC) than 60NiTi (~60 HRC) in hardened conditions. In essence, once hardened, a lesser amount of nanoscale Ni_4_Ti_3_ is expected to be formed in the NiTi matrix of these parts as compared to the ideal amount formed in that of 60NiTi, leading to the activation of an Orowan strengthening mechanism [1]. In addition, it can be interpreted that a higher hardness value could be expected to be obtained for the 60NiTi parts processed in this research, if the applied hardening treatment was successful in a full dissolution of Ni_3_Ti and Ni_3_Ti_2_ particles causing the maximum precipitation of nanoscale Ni_4_Ti_3_ in their NiTi matrix.

In essence, this research, despite not being successful in fully processing dense 58NiTi and 60NiTi parts, provides a guideline for the production of Ni-rich NiTi alloys from elementally blended Ni–Ti powder mixtures. The obtained results help to predict the microstructural and phase compositional evolution of parts that are printed from elementally blended Ni and Ti powders and are next thermally treated. Moreover, the results of this research provide information on what might not work or be challenging when processing Ni-rich NiTi parts from elemental Ni and Ti powders. These should be useful to other researchers working on essential studies that are focused on the development of lower-cost processes helping the wide exploitation of these nickel-rich NiTi structural alloys.

## 3. Materials and Methods

### 3.1. Powder Characterization

The commercially gas atomized elemental Ti (D90 = 86.2 µm) and Ni (D90 = 43.3 µm) powders, purchased, respectively, from Beijing Xing Rong Yuan Technology Co., Ltd., Beijing, China and Chengdu Huayin Powder Technology Co., Ltd., Chengdu, China, were used to prepare two different batches of Ni–Ti elemental powder mixtures. These prepared powder mixtures, having about 60 wt.% Ni + 40 wt.% Ti and 58 wt.% Ni + 42 wt.% Ti in their compositions were used to, respectively, print 60NiTi and 58NiTi parts.

O, H, C, S, and N impurities existing in the composition of mixed powders were determined by inert gas fusion analytical instrument Leco machines. These tests were performed for three different powder batches and the average results are reported.

The morphology of the elemental powders (based on the emissions received from the secondary electrons) in their original purchased conditions was characterized by a JXA-8100 electron probe microanalyzer (EPMA) machine equipped with an energy dispersive X-ray spectrometer (EDS). The values of the two different powder mixtures’ particle diameters at 10%, 50% and 90% in the cumulative distribution were determined using a laser diffraction particle sizing measurement machine (Malvern Mastersizer 3000, Malvern Panalytical, Malvern, United Kingdom). Hall flowability, apparent density, and tap density of the powder mixtures were investigated by ASTM B213, ASTM B964, ASTM B212, and ASTM B527 standards, respectively [48]. Note that tests concerning particle size distribution, Hall flowability, Carney flowability, apparent density, and tap density were performed on three different powder batches and the average results are reported.

### 3.2. LPBF Processing and HIP Treatment

A commercial BLT-A100 LPBF machine, Xi’an Bright Laser Technologies, Xi’an, China was employed to print cubic samples with 12 mm height and a 6 × 6 mm square cross-section (CAD model). Various sets of printing parameters, covering different laser powers and scanning velocities, were used to print both the 58NiTi and 60NiTi parts. During the sample processing and regardless of the used set of processing parameters, the orientation of hatches was changed by 60° for successive layers. The fabrication chamber of the used machine, equipped with a Yb fiber laser with a wavelength of 1070 nm and a beam spot size of 60 µm in diameter, was continuously purged with the Ar/N2 gas atmosphere. As a result of this, the amount of O was kept at less than 0.01 wt.% during the fabrication process. A near-equiatomic NiTinol plate was used in this study as the build platform to print the powders on and was heated to 180 °C during the process. These were executed to decrease the possibility of delamination or cracking of samples having roots in the generated thermal stresses that are caused by the difference existing between the thermal expansions of the build platform and the printed parts [31,36]. A total of 8 60NiTi and 58NiTi samples were printed by each specific set of processing parameters.

A QIH-15L Quintus HIP machine was next used to postprocess the samples printed using selected sets of printing parameters. Considering the capability of this machine, samples can be treated by a combined maximum pressure of 200 MPa and a temperature of 1400 °C. In this study, HIP treatments were performed at different designed temperatures and for different holding times but under a constant pressure of 180 MPa. Details regarding these processes consisted of vacuuming the furnace of the machine to below 950 Pa and then, as the machine was heating (20 °C/min) the samples from ambient temperature to different design temperatures, increasing the pressure applied by the Ar gas, acting also as a protecting gas, in a gradual mode to 180 MPa. The processed samples were then naturally cooled inside the machine once the HIP treatment procedures were finished. As, during the operation, the HIP’s furnace temperature experiences about +2 °C deviation from the temperature designed to treat the parts, the actual temperatures recorded by the thermocouples in the furnace are reported in this manuscript as the actual treating temperatures.

The final typical hardening treatment performed on the processed parts consisted of heating the parts to ~1000 °C using a muffle furnace, holding the samples at this temperature for an hour, and then quenching them in water resulting in fast cooling rates [7,49].

### 3.3. Characterization of Processed Parts

The dimensional integrities of the as-printed and post-HIP processed samples were evaluated by comparing the geometry of the as-printed samples with that of the CAD file and also comparing the geometry of samples before and after doing the HIP procedures. The Archimedes method, as specified in the ASTMB962 standard, was used to measure the density of the samples [50]. For each of the processed parts, these experiments were performed on five different samples and the average results are reported. In addition, the Leco machines mentioned before were used to determine the amounts of impurities such as O, H, C, S, and N existing in the composition of the top side of the processed parts. These tests were performed three times and the average results are reported.

The microstructural images of parts (based on the emissions received from the secondary electrons) were obtained using the mentioned EPMA machine. The phase constituents of parts were identified by a combination of a RIGAKU high-resolution X-ray diffractometer (XRD), using copper Kα radiation, and the EDS system providing information on elemental distribution and the elemental ratio of different precipitates existing at different spots and regions of the cross-section of samples. The Ni:Ti atomic ratio reported for each specific NiTi intermetallic phase in [22] was used to interpret the data obtained by the EDS and identify the phases in the microstructure of the processed parts. Note that these tests were performed on the cross-section of the ground, polished, and etched samples and the images were obtained in parallel to the building direction (BD) of parts. The receipt for the preparation of the Ni-rich NiTi parts for microstructural studies is discussed and explained in [40,41,42,44]

A Rockwell hardness tester, using a conical diamond indenter applying 150 Kg load, was used to measure the Rockwell C hardness (HRC) of the processed parts. These tests were performed 10 times and the average results are reported. Note that the HRC tests were performed on the ground and polished cross-section of the samples, prepared with a procedure identical to that used for the microstructural studies.

## 4. Conclusions

The purpose of this research was to process dense 60NiTi and 58NiTi parts having homogeneous microstructures from Ni–Ti powder mixtures with about 60 wt.% and 58 wt.% Ni, respectively. The prepared powder mixtures were, at the first stage, printed by an LPBFF method using different processing parameters. In the next step, parts, printed with selected printing sets, were treated by elementary and secondary HIP treatments to homogenize the microstructures and eliminate their existing cracks and pores.

The following conclusions were obtained:

1. The printability of powder mixtures was reduced under the conditions that the laser powers of above 50 watts were employed to print the parts. In addition, for the sets of processing parameters having a laser power of 50 watts, the powders showed the best printability with the set of processing parameters (set 1) having the lowest laser energy input (27.70 J/mm^3^). Set 4, having a laser power of 50 watts, was the highest used energy input (39.68 J/mm^3^) that the powder mixtures showed an almost reasonable printability with.
2. Due to the effect of the melt pool temperature and generated residual stresses, the more Ni-rich powder mixture, with about 60 wt.% Ni and 40 wt.% Ti in its composition showed a relatively better printability than the one with about 58 wt.% Ni and 42 wt.% Ti.
3. Microstructures of the parts printed by these processing sets, regardless of the composition of the used powder mixtures, were inhomogeneous and consisted of not fully reacted Ni-rich and Ti-rich areas. Due to the differences existing in the energy inputs of the printing sets causing different levels of residual stresses and diffusion amongst printed layers, microstructures of parts printed by set 1 were dominant with large pores and the ones with set 4 with cracks.
4. Applying an elementary HIP treatment at 1050 °C for 4 h, homogenized the as-printed parts and microstructures conforming to those of 58NiTi and 60NiTi alloys were obtained. However, the applied treatments could not eliminate the cracks and pores existing in the microstructures of as-printed parts. The average relative densities of the parts obtained from samples printed by set 4 (ranging between ~96 and ~98%) were relatively higher than those processed from parts printed by set 1 (ranging between ~93 and ~94%).
5. Secondary HIP treatments were designed to eliminate these flaws from the microstructures of elementary HIP-treated parts. However, this aim could not be achieved under conditions that the applied HIP temperatures were below the ones at which the dimensional integrities of parts were deteriorated.
6. Regardless of the composition of the used powder mixtures and processing sets used to print the parts, the average apparent hardness (ranging between ~38.8 and ~46.1 HRC) and relative densities (ranging between ~94 and ~97%) of the secondary HIP-treated parts, performed at the maximum temperatures at which the dimensional integrities of the parts were respected, were negligibly different as compared to the average apparent hardness (ranging between ~42.3 and ~44.8 HRC) and relative densities of elementary HIP-treated ones (ranging between ~93 and ~98%).
7. Applying the final hardening treatment on the secondary HIP treated parts, processed from samples printed by set 1, resulted in a significant drop of the average apparent hardness (ranging between ~38.9 and ~42.4 HRC) and relative densities (ranging between ~94 and ~95%) to ranges between ~19.6–~26.1 HRC and ~83–~84%, respectively. The amounts and sizes of the pores existing in the microstructures of these secondary HIP-treated parts were significantly increased due to the effects caused by the conduction of this treatment.
8. The 60NiTi parts showed a higher hardening potential than the 58NiTi ones. The applied hardening treatments decreased the relative density of secondary HIP-treated 58NiTi and 60NiTi parts, processed from samples printed by set 4, from around ~97% to a range between ~93 and ~94%. These treatments led to the widening of cracks that existed in the microstructures of the secondary HIP-treated parts. The obtained average hardness values of the hardened 58NiTi parts (~35.8 HRC) were decreased insignificantly as compared to those of secondary HIP-treated 58NiTi ones (~46.1 HRC), due to the effects caused by the hardening treatment. However, the hardening treatments, despite not being able to fully dissolve the Ni-rich Ni_3_Ti and Ni_3_Ti_2_ phases existing in the microstructure of furnace-cooled 60NiTi samples, led to the increase in hardness values of 60NiTi parts from ~38.8 HRC to ~45.9 HRC.

## Figures and Tables

**Table 1:** Some physical properties of mixed powders having about 60 wt.% Ni + 40 wt.% Ti in its composition.

Particle Size Distribution (µm)	Hall Flowability (s/50 g)	Apparent Density (g/cm^3^)	Tap Density (g/cm^3^)
D90 = 47.30 D50 = 33.10 D10 = 23.8	16.50	4.03	4.38

**Table 2:** Amounts of impurities existing in the powder mixtures having about 60 wt.% Ni + 40 wt.% Ti and 58 wt.% Ni + 42 wt.% Ti in their compositions.

Powder Mixtures	O (wt.%)	N (wt.%)	S (wt.%)	C (wt.%)	H (wt.%)
60 wt.% Ni + 40 wt.% Ti	~0.0374	~0.0034	~0.0008	~0.0038	~0.0009
58 wt.% Ni + 42 wt.% Ti	~0.0380	~0.0035	~0.0007	~0.0039	~0.0009

**Table 3:** Different sets of LPBF processing parameters used to print the cubic samples from prepared powder mixtures having about 60 wt.% Ni + 40 wt.% Ti and 58 wt.% Ni + 42 wt.% Ti compositions.

Printing Sets	Laser Power (W)	Scanning Velocity (mm/s)	Hatch Spacing (mm)	Layer Thickness (mm)	Volumetric Laser Energy Input (J/mm^3^)	60 wt.% Ni + 40 wt.% Ti Powder-Number of Printed Samples (out of 8)	58 wt.% Ni + 42 wt.% Ti Powder-Number of Printed Samples (out of 8)
**1**	50	500	0.12	0.03	27.70	8	8
**2**	50	450	0.12	0.03	30.86	8	7
**3**	50	400	0.12	0.03	34.72	8	7
**4**	50	350	0.12	0.03	39.68	7	6
**5**	50	300	0.12	0.03	46.29	5	3
**6**	50	250	0.12	0.03	55.55	0	0
**7**	50	200	0.12	0.03	69.44	0	0
**8**	60	500	0.12	0.03	33.33	6	6
**9**	60	400	0.12	0.03	41.66	3	0
**10**	60	300	0.12	0.03	55.55	0	0
**11**	60	250	0.12	0.03	66.66	0	0
**12**	70	450	0.12	0.03	43.20	0	0
**13**	90	600	0.12	0.03	41.66	0	0

**Table 4:** Table showing the relation existing amongst the temperatures of applied secondary HIP treatments and dimensional integrities of processed 60NiTi and 58NiTi parts. Note, the highlighted parts show the temperatures at which the dimensional integrities of parts were respected.

Samples	Printing Sets Used to Process As-Printed Parts	Secondary HIP Treatment Temperatures	Conditions of Samples
**60NiTi**	1	1180 °C	Sample was melted
		1175 °C	Sample was melted
		1170 °C	Dimensional integrity affected significantly
		1167 °C	Dimensional integrity affected significantly
		1165 °C	Dimensional integrity respected
**60NiTi**	4	1180 °C	Sample was melted
		1175 °C	Sample was melted
		1170 °C	Dimensional integrity affected significantly
		1167 °C	Dimensional integrity affected significantly
		1165 °C	Dimensional integrity respected
**58NiTi**	1	1180 °C	Dimensional integrity affected lightly
		1177 °C	Dimensional integrity affected lightly
		1175 °C	Dimensional integrity respected
**58NiTi**	4	1180 °C	Sample was melted
		1177 °C	Dimensional integrity affected significantly
		1175 °C	Dimensional integrity affected significantly
		1172 °C	Dimensional integrity affected lightly
		1170 °C	Dimensional integrity respected

**Table 5:** Average relative densities and apparent hardness values of 58NiTi and 60NiTi parts processed by elementary HIP treatments from parts printed by sets 1 and 4. Average relative densities and apparent hardness values of secondary HIP-treated parts and hardened ones are also reported in this table.

Sample	Average Relative Density (%)	Apparent Hardness (HRC)	Standard Deviation of Measured Hardness Values
58NiTi, set 1 (Elementary HIP treated)	~94	~44.8	~3.1
58NiTi, set 1 (Secondary HIP treated at 1175 °C)	~95	~42.4	~3.4
58NiTi, set 1 (Hardened)	~83	~19.6	~5.8
58NiTi, set 4 (Elementary HIP treated)	~96	~43.1	~3.5
58NiTi, set 4 (Secondary HIP treated at 1170 °C)	~97	~46.1	~3.2
58NiTi, set 4 (Hardened)	~93	~35.8	~4.4
60NiTi, set 1 (Elementary HIP treated)	~93	~42.4	~5.1
60NiTi, set 1 (Secondary HIP treated at 1165 °C)	~94	~38.9	~4.4
60NiTi, set 1 (Hardened)	~84	~26.1	~6.7
60NiTi, set 4 (Elementary HIP treated)	~98	~42.3	~1.4
60NiTi, set 4 (Secondary HIP treated at 1165 °C)	~97	~38.8	~1.8
60NiTi, set 4 (Hardened)	~94	~45.9	~2.1

**Table 6:** Amounts of N and O impurities existing in the compositions of samples at different stages of as-printed, elementary HIP-treated, and secondary HIP-treated. These samples were processed from parts printed by set 4, having an energy input of 39.68 J/mm^3^, using the 58 wt.% Ni and 42 wt.% Ti powder mixture.

Samples	O (wt.%)	N (wt.%)
As-printed by set 4 from 58 wt.% Ni and 42 wt.% Ti powder mixture	~0.0405	~0.0037
Elementary HIP treated	~0.0455	~0.0195
Secondary HIP treated at 1170 °C	~0.0675	~0.023

## Data Availability

The datasets generated during and/or analyzed during the current study are available from the corresponding author on reasonable request.
